# A validation study of the Korean version of the Toronto empathy questionnaire for the measurement of medical students’ empathy

**DOI:** 10.1186/s12909-021-02561-7

**Published:** 2021-02-19

**Authors:** Sanghee Yeo, Kyong-Jee Kim

**Affiliations:** 1 Department of Medical Education, School of Medicine, Kyungpook National University, Daegu, South Korea; 2 Department of Medical Education, Dongguk University School of Medicine, 32 Dongguk-ro, Ilsandong-gu, Goyang-si, Gyeonggi-do 10326 South Korea

**Keywords:** Empathy, Medical student, Psychometrics, Toronto empathy questionnaire

## Abstract

**Background:**

This study aimed to validate the Korean version of the Toronto Empathy Questionnaire (TEQ) and to determine its suitability for the measurement of empathy in medical students.

**Methods:**

The study sample was Year 1 and 2 medical students at two medical schools on six-year undergraduate medical programs in South Korea. The study participants completed the Korean TEQ, which has a single factor structure and consists of 16 items; responses are scored using a 5-point Likert scale, giving a maximum possible score of 64. Psychometric validation of the questionnaire was performed by exploratory and confirmatory factor analyses and the goodness of fit test. Average variance extracted was calculated to establish convergent validity, and associations between factors and construct reliability were analyzed to establish discriminant validity. Cronbach’s alpha values were utilized for reliability analysis.

**Results:**

A total of 279 students completed and returned the questionnaire (a 96.2% response rate). Participant empathy scores ranged from 20 to 60 (M = 44.6, SD = 7.36). Empathy scores were higher for females than males (*p* < .05). The cumulative variance of the Korean TEQ was 32%, indicating that its explanatory power was rather weak. Consequently, goodness-of-fit testing was performed on four hypothetical models, among which a three-factorial structure consisting of 14 items demonstrated satisfactory fit indices and explained 55% of the variance. Reliability estimates of the three subscales were also satisfactory (Cronbach’s α = .71–.81). This three-factorial model was validated by confirmatory factor analysis and demonstrated adequate convergent and discriminant validity.

**Conclusions:**

This study demonstrated psychometric validation of the Korean TEQ for measuring medical students’ empathy. We suggest a modified 14-item model with a three-factorial structure, which demonstrated better psychometric properties than the original scale.

## Background

Empathy is defined as the ability to share or understand the emotional state of others. In a clinical setting, empathy encompasses the ability to appreciate patients’ emotions and to express this awareness to patients [1]. Empathy is recognized as a key professional competency for healthcare professionals [2–5], and thus is known to be an important attribute for medical students. Research indicates medical student burnout, professionalism, and personality attributes that affect interpersonal relationships are associated with empathy [6, 7]. Therefore, it is important to measure medical student empathy and offer them appropriate interventions to promote their development of professional competencies. Hence, past studies of empathy in medical education have focused on assessing medical student empathy skills and on the psychometric properties of such instruments [8].

There are several instruments for measuring empathy that are well known and widely used in medical education. The Jefferson Scale of Physician Empathy (JSE) is one of the most widely used instruments to measure medical student empathy [9], and it is known as a valid and reliable tool [10]. JSE was used in a nationwide study of empathy of Korean medical students [11] and it was found that their empathy scores were lower than those of their Western counterparts. The researchers [11] related the lower empathy scores of Korean medical students to the Korean cultures that they are less dependent on non-verbal communication and that they regard it as a virtue to have a calm, unemotional, and less assertive attitude.

The Interpersonal Reactivity Index (IRI) inventory has also been widely used in medical education research [12]. The IRI is a self-report instrument for measuring empathy in general populations, and its constructs encompass cognitive and emotional dimensions of empathy, which comprises four subscales, that is, perspective taking, fantasy, empathetic concern, and personal distress [13]. The IRI was translated into Korean by Kang and colleagues, and they found the Korean IRI was valid and reliable in a study of general populations and medical students in Korea [14]. Still, Kang and colleagues [14] did not focus on medical students in their study of the Korean IRI, in which they blended medical students with general populations in their study sample; thus, their study lacks in sufficient evidence for suitability of it use for Korean medical students. Furthermore, it has been argued that IRI has items that measure personal qualities other than empathy [15].

The Toronto Empathy Questionnaire (TEQ), which was developed and validated by Spreng and colleagues [15] is another well-known tool for measuring empathy. The TEQ is devised to target general populations and represents empathy primarily as an emotional process [15]. Although the TEQ has been studied in medical students in international settings [16–18], its psychometric properties have seldom been investigated in non-English-speaking samples [18]. The Korean version of the TEQ was derived by translation and validated in a study of approximately 500 Korean undergraduates and graduate students by Kim and Han [19], but has not yet been validated in a population of Korean medical students.

Assessing empathy of medical students during the early study years has particular importance in the context of Korean medical schools, most of which have a six-year basic medical education program with an initial 2-year premed curriculum. As it is an educational goal of premed programs to develop professional competencies expected of good doctors, fostering medical student empathy is often emphasized. Still, few studies have addressed measurements of empathy suitable for medical students during the early years. Therefore, there is a need for empathy measurement tools in non-clinical contexts for medical students during the early phase of their curricula and for studies on its psychometric properties.

Although the JSE and the IRI are widely used in medical education, research indicates that the two instruments are only weakly related, which suggests they may measure different constructs [10, 12]. Despite the various assessment tools available for measuring medical student empathy, the literature has shown weak relations among different measurement tools and has also shown mixed results in their stability over time during basic medical education [20, 21]. Therefore, research is needed to provide a more valid measure of empathy among medical students. Moreover, although JSE and IRI have been used to measure medical student empathy, there is no study of suitability of using TEQ to measure medical student empathy in the Korean context. Thus, we aimed to validate the Korean TEQ and to investigate its suitability for measuring empathy in medical students in very early years.

## Methods

### Study settings and participants

The study participants were first- and second-year medical students enrolled at two South Korea medical schools. One (KNUSOM) is a national medical school located in a metropolitan area with an approximate annual intake of 110 students, and the other (DUSOM) is a private medical school located in a mid-sized city with an approximate annual intake of 50 students. Both of these medical schools operate six-year undergraduate-entry medical programs, in which premed courses are offered during the first 2 years. None of the participants were exposed to formal training on empathy in their medical education programs prior to this study. The sample size for this study was 290, which met the minimum requirement suggested by Kang [22] that there is a low risk of distorting results when a sample size of ≥200 subjects is used in factor analysis studies.

### The research instrument

The Korean version of the TEQ was used in this study. The Korean TEQ was translated by Kim and Han [19], who validated it in a population of undergraduate and graduate students and found it to have adequate psychometric properties [19]. The original and the Korean version of the TEQ were validated as single-factor models by exploratory factor analysis [15, 19]. The Korean TEQ consisted of 16 items, which given the limitations of translation are identical to those of the original instrument. The items were rated using a 5-point Likert scale ranging from 0 (“This sentence does not describe me very well”) to 4 (“This sentence describes me best”). The questionnaire contains 8 items with negative connotations (#2, 4, 7, 10, 11, 12, 14, and 15), which were reverse coded for the analysis. Empathy scores range from a possible maximum of 64 to a minimum of 0, where a higher score indicates better empathic ability.

We obtained permission to use the Korean TEQ from the lead researcher who had translated and validated it via e-mail. By using a questionnaire that had already been translated into Korean, we hoped to reduce translation-related validity degradation issues.

### Data collection and ethical considerations

Questionnaires were administered in a paper-based format from March to April, 2019 after acquiring permission from the Institutional Review Board of Dongguk University School of Medicine (DGU IRB 20190013). The researchers provided all participating students with a description of the purpose and methods of the study, stressed their rights regarding voluntary participation in the study, and assured them of personal confidentiality. Students who agreed to participate in the survey completed the questionnaire, and a student representative collected responses and submitted them to the researchers.

### Data analysis

The general characteristics of participants, test items, and empathy scores were analyzed using descriptive statistics. To check for normal distribution of data, we analyzed data skewness and kurtosis and performed the Kolmogorov-Smirnov test. The Mann-Whitney test was conducted to investigate differences among the demographic variables (e.g., gender, age, grade, and school).

Exploratory factor analysis was performed to investigate the factor structure, and this was followed by reliability analysis using Cronbach’s alpha values. The psychometric properties of the Korean TEQ were examined in terms of its validity, reliability, and goodness of fit. Validity testing generally includes tests for content validity, construct validity, convergent validity, discriminant validity, and criterion-related validity. As we aimed to validate the existing instrument, we did not perform content validity testing and focused on estimating construct validity.

Confirmatory factor analysis was performed to investigate the suitability of the single factor model proposed by the original authors and also to investigate construct validity. Confirmatory factor analysis was performed by direct oblimin rotation after applying the maximum likelihood method. Average Variance Extracted (AVE) was calculated to establish convergent validity. To estimate discriminant validity, correlation coefficients were calculated between factors constructing the instrument. Cronbach’s alpha values were calculated to establish the internal consistency of items.

The analysis was performed using SPSS 25.0 and AMOS 26, and statistical significance was accepted for *p* values of < .05.

## Results

A total of 279 students completed and returned the questionnaire, a 96.2% response rate. All 99 students from DUSOM (100%) completed the questionnaire, and 181 out of 191 from KNUSOM (94.8%) did so.

### Participant demographics and empathy scores

Participant demographics and empathy scores are provided in Table 1. Of the 279 participants, 178 were male (63.8%) and 101 were female (36.2%). Forty-eight percent of the participants (*n* = 133) were aged 18 to 19, and the remaining 52.3% (*n* = 146) were aged ≥20. The age distribution ranged from 18 to 24 years (M = 19.72, SD = 1.05).

**Table 1:** Participant demographics and empathy scores

Variable		N (%)	M ± SD	p*
Year	1st	159 (57.0)	44.3 ± 7.48	0.49
	2nd	120 (43.0)	44.9 ± 7.21	
Gender	Male	178 (63.8)	43.9 ± 7.59	0.04
	Female	101 (36.2)	45.8 ± 6.81	
Age	18–19 years	133 (47.7)	44.8 ± 7.58	0.60
	Above 20	146 (52.3)	44.4 ± 7.18	
School	DUSOM	99 (35.5)	44.7 ± 7.27	0.96
	KNUSOM	180 (64.5)	44.5 ± 7.44	
Total		279 (100.0)	44.6 ± 7.36	

* *p*-values were calculated using the Mann-Whitney U test

Participant empathy scores ranged from a minimum of 20 to a maximum of 60. Mean empathy score of the participants was 44.6 (SD = 7.36) and median score was 45.0. Female students had higher empathy scores than male students (*p* < .05). Ages, years in medical school, and institution had no effect on empathy scores.

Descriptive statistics of the items in the Korean TEQ are provided in Table 2. Skewness and kurtosis for each of the 16 items in the questionnaire ranged from −.23 to − 1.43 and from −.66 to 4.08, respectively. The skewness and kurtosis of total empathy scores were − .32 (se = .15) and − .26 (se = .3), respectively. The Kolmogorov-Smirnov test confirmed that data were not normally distributed (*p* < .01). Thus, it was decided that non-parametric tests be used for the statistical analysis. Cronbach’s alpha co-efficient of the Korean TEQ was .855, which demonstrated a high level of internal consistency.

**Table 2:** Descriptive statistics of items in the Korean Toronto Empathy Questionnaire (TEQ)

item	M	SD	Skewness	Kurtosis
1. When someone else is feeling excited, I tend to get excited too.	3.13	0.83	−1.02	1.28
2. Other people’s misfortunes do not disturb me a great deal.	2.95	0.89	−0.85	0.76
3. It upsets me to see someone being treated disrespectfully.	3.37	0.79	−1.68	4.08
4. I remain unaffected when someone close to me is happy.	2.92	0.84	−0.74	0.43
5. I enjoy making other people feel better.	3.06	0.77	−0.58	0.30
6. I have tender, concerned feelings for people less fortunate than me.	2.97	0.82	−0.78	0.83
7. When a friend starts to talk about his/her problems, I try to steer the conversation towards something else.	3.38	0.78	−1.43	2.57
8. I can tell when others are sad even when they do not say anything.	2.70	0.87	−0.42	−0.09
9. I find that I am “in tune” with other people’s moods.	2.94	0.83	−0.57	0.09
10. I do not feel sympathy for people who cause their own serious illnesses.	2.41	1.06	−0.32	−0.66
11. I become irritated when someone cries.	3.06	0.92	−0.74	−0.01
12. I am not really interested in how other people feel.	2.73	1.04	−0.59	−0.44
13. I get a strong urge to help when I see someone who is upset.	2.28	0.97	−0.23	−0.45
14. When I see someone being treated unfairly, I do not feel very much pity for them.	3.27	0.75	−1.06	1.62
15. I find it silly for people to cry out of happiness.	3.38	0.83	−1.38	1.75
16. When I see someone being taken advantage of, I feel kind of protective towards him/her.	2.75	0.84	−0.45	0.12
Total	44.58	7.36	−0.32	−0.26

Notes: Responses to items 2, 4, 7, 10, 11, 12, 14, and 15 (negatively worded items) were reversed coded to calculate means and total scores

### Factor structure

Table 3 shows item-total correlations and factor loadings obtained by exploratory factor analysis. The correlation coefficients of items 3, 4, and 15 were .159, .282, and .286, respectively. Items 3 and 4 had the absolute value of factor loading lower than .4 (.210 and .379, respectively), but item 15 had factor loading greater than .4 (.499). The Kaiser-Meyer-Olkin (KMO) value was .862, and Bartlett’s sphericity test showed *χ*^2^ = 1310.523 (*p* < .001), which confirmed that the original 16-item model was suitable for factor analysis. However, the cumulative variance amounted to 32.165%, indicating that the explanatory power of the Korean TEQ was rather weak.

**Table 3:** Item-total correlation, communality and factor loadings of the Korean TEQ

Item	Item-total correlation	Cronbach’s α if item deleted	Communality	Factor loadings
1	.426	.849	.424	.676
2	.508	.845	.427	.645
3	.353	.852	.159	.210
4	.478	.847	.282	.379
5	.522	.845	.445	.605
6	.497	.846	.343	.488
7	.449	.848	.794	.919
8	.419	.850	.387	.633
9	.491	.846	.407	.592
10	.435	.850	.354	.612
11	.504	.845	.443	.528
12	.636	.837	.488	.431
13	.503	.845	.451	.657
14	.634	.840	.492	.523
15	.352	.853	.286	.499
16	.470	.847	.344	.556

Subsequently, factor analysis was carried out using 14 items, that is, excluding items 3 and 4, which had been found to degrade the validity of the scale during earlier analysis. As a result, the factor loading of all items rose to 0.5 or higher. The KMO value of the 14-item model was .858, and Bartlett’s sphericity test showed *χ*^2^ = 1143.002 (*p* < .001), indicating that the goodness-of-fit of the model had been maintained. Furthermore, the cumulative variance increased to 54.999% when the model comprised three factors, which indicated an improved explanatory power. The Cronbach’s alpha coefficient of the 14 items was .844, which demonstrated high internal consistency.

Given that the original Korean TEQ was a single-factor model and that goodness-of-fit was maintained when items 3 and 4 were removed, we performed confirmatory factor analyses and goodness of fit tests on the following four models: (a) a single-factor model with 16 items, (b) a 3-factor model with 16 items, (c) a single-factor model with 14 items, and (d) a 3-factor model with 14 items.

### Goodness of fitness of the models and confirmatory factor analysis

Goodness of fit statistics of the four hypothetical models are summarized in Table 4. In terms of Minimum Discrepancy per Degree of Freedom (CMIN/DF), a model should have a value < 3.0, and for Normed Fit Index (NFI), it is considered that a larger value provides better model fit [23]. Models 2 and 4 had a high level of goodness-of-fit with CMIN/DF values of < 3.0 and NFI values of ≥ .09. Turker Lewis Index (TLI) and Comparative Fit Index (CFI) indicate acceptability when > .90 [23]. Whereas TLI values were below acceptable levels in all four models, Model 4 reached the standard CFI value. RMSEA (Root Mean Square Error of Approximation), which is a measure of overall goodness-of-fit, values were < .08 in Models 2 and 4, which were at acceptable levels [24]. Cronbach’s α of the three factors were .81 (Factor 1), .73 (Factor 2), and .71 (Factor 3), which were also at acceptable levels.

**Table 4:** Goodness of fit statistics of the four hypothetical models

Model	χ^2^ (*p*)	CMIN/DF	NFI	TLI	CFI	RMSEA
1	412.179 (< .001)	3.963	.692	.709	.747	.103
2	240.313 (< .001)	2.379	.821	.864	.886	.070
3	344.121 (< .001)	4.469	.705	.706	.752	.112
4	170.624 (< .001)	2.306	.854	.889	.910	.069

Notes: Model 1 = 16 items with one factor, Model 2 = 16 items with 3 factors, Model 3 = 14 items with one factor, Model 4 = 14 items with 3 factors

*CMIN/DF* Minimum Discrepancy per Degree of Freedom, *NFI* Normed Fit Index, *TLI* Turker Lewis Index, *CFI* Comparative Fit Index, *RMSEA* Root Mean Square Error of Approximation

Confirmatory factor analysis was performed on Model 4. Bartlett’s test of sphericity was significant (*χ*^2^ = 170.624, *p* < .001). This model demonstrated a high level of goodness-of-fit, where CMIN/DF = 2.306, NFI = .854, TLI = .889, CFI = .910, RMSEA = .069. Therefore, Model 4 yielded satisfactory fit indices.

### Validity of the instrument

#### Convergent validity

Standardized regression coefficients calculated for Model 4 are provided in Table 5. All 14 items were significantly associated with the three factors (*p* < .001). It is generally considered that convergent validity is adequate when the AVE value exceeds .05 [25]. The convergent validities of Factors 2 and 3 were acceptable with AVE values of ≥ .05, while convergent validity of Factor 1 was relatively weak.

**Table 5:** Convergent validity of items

Item No.	Factors	Standardized Estimate	Estimate	Standard Error	Critical Ratio	*p*	AVE^a^	Construct reliability
12	1	.70	.54	.06	9.44	< .001	.42	.84
6		.56	.46	.04	10.70	< .001		
14		.71	.27	.03	9.37	< .001		
16		.57	.47	.04	10.64	< .001		
10		.52	.82	.07	10.89	< .001		
2		.63	.48	.05	10.26	< .001		
13		.60	.59	.06	10.38	< .001		
9	2	.66	.39	.04	8.66	< .001	.50	.80
5		.66	.34	.04	8.73	< .001		
8		.62	.46	.05	9.34	< .001		
1		.59	.44	.04	9.75	< .001		
11	3	.72	.40	.06	6.98	< .001	.55	.78
15		.56	.47	.05	9.97	< .001		
7		0.75	.27	.04	6.49	< .001		

^a^*AVE* Average Variance Extracted

#### Discriminant validity

To establish the discriminant validity of a model, it has been suggested that the AVE of each of the constructs in a model be greater than the square of the correlation between the constructs [25]. Table 6 presents correlation coefficients between factors. The square of the correlation coefficients between all constructs fell within the range between .18 and .40. Since the square of correlation coefficients was < .42, which is the smallest AVE value of the constructs, all constructs in Model 4 were deemed to demonstrate adequate discriminant validity.

**Table 6:** Correlation coefficients between factors

Factors	Correlation coefficients (*r*^2^)		Average Variance Extracted	Construct reliability
	1	2		
1	1		.42	.84
2	.63 (.40)*	1	.50	.80
3	.58 (.34)	.43 (.18)*	.55	.78

*p < .001

## Discussion

Our study showed the empathy scores of Korean medical students obtained using the Korean TEQ were similar to those reported in a study of US college students [15] and were lower than those reported for Caribbean and Malaysian medical students [16, 17]. These findings seem to indicate the empathy levels of Korean premed students are similar with those of college students but are lower than those of medical students in other countries. Still, Korean medical students’ TEQ scores were slightly higher than those of Chinese medical students [18]. The lower TEQ scores of Chinese and Korean medical students likely pertain to cultural influences on empathy, which were mentioned earlier, as these two countries have similar cultural backgrounds. Our study also found that female students demonstrated higher empathy scores than males, which concurs with the findings of other studies that showed gender differences in medical students [11, 20, 26]. Our findings add to knowledge regarding the validity of the TEQ in an international context.

The present study demonstrates that the Korean TEQ has adequate psychometric properties. However, we found this questionnaire was more valid when two items were excluded and three constructs were used than the single-factor model of the original instrument. These three factors comprised of 14 items accounted for 55% of the variance, which demonstrated better exploratory power than the original instrument as reported by Kim and Han [19]. We suggest that the three-factor model is a better proposition than the single factor model as it better fits the guideline for factor analysis that total variance explained should be more than 60 % for a construct to be valid [27]. Moreover, the three-factor model yielded satisfactory fit indices by confirmatory factor analysis and showed acceptable reliability levels. This model also demonstrated adequate convergent and discriminant validity. Therefore, we suggest that the modified Korean TEQ with three factors using 14 items is more valid and parsimonious for assessing medical student empathy during in the early phase of medical education.

In our study, seven of the 14 items retained in the three-factor model loaded on the first factor (items 13, 2, 10, 16, 14, 6, 12), the second factor included four items (1, 8, 5, and 9), and three items (7, 15 and 11) loaded on the third factor. Based on the items composing the factors, we suggest that the first factor be labeled as “Having sympathy for others”, the second factor as “Recognizing the feelings of others”, and the third factor as “Avoiding resonating with the feelings of others.” These three constructs seemingly share similarity with those of the IRI, as they can be paired with empathetic concern, perspective taking, and personal distress, respectively.

Although previous studies have investigated associations between the IRI and JES [10, 12], research is scant on the relationship between IRI and TEQ in the context of medical students. This finding is in line with the findings from previous studies that show associations between IRI and TEQ in the study of general populations [15, 19]. This finding may be due, in part, to the fact that the two instruments share similar constructs as they focus on emotional dimensions of empathy. Future research is warranted for further investigation of the relationship between the underlying constructs of these two instruments.

The present study was the first Korean study to validate the TEQ for the assessment of empathy in medical students. Furthermore, our study came up with a 3-factor model that showed a higher level of explanatory power than the original scale by confirmatory factor analysis. As Kim and Han [19] pointed out, Spreng et al. [15] reported unsatisfactory values for some fit indices as determined by exploratory factor analysis and did not perform confirmatory factor analysis in their validation study of TEQ. Our findings support the assertion made by Kim and Han [19] of the possibility of a non-single factor model of the Korean TEQ. Our finding is also consistent with that of Xu and colleagues [18] that supported a 3-factor structure of the Chinese TEQ.

Several limitations of the present study should be acknowledged. First, we used a self-report measure and it was beyond the scope this study to investigate the nature of its relationship with other measurements of empathy. Past studies have indicated weak associations between medical student empathy scores using self-report measures and those using other measures, such as observation by faculty or ratings by standardized patients [28–30]. Thus, future research is recommended to investigate relationships between medical student self-reported empathy using the Korean TEQ and other measures of empathy for further validation of the instrument. Second, as TEQ is targeted for general population, it is yet to see whether it has predictive validity for clinical empathy. As Hemmerdinger [8] pointed out, no empathy measures offer sufficient evidence of predictive validity for medical students. This warrants future research of predictive validity of TEQ for medical students. Third, we used the empathy scale primarily focused on the emotional dimensions in this study. Still, the literature indicates empathy is multidimensional and various constructs have been suggested to define it [10, 12]. Therefore, future research is warranted to better understand relationships between measures of empathy that use other constructs to better understand the validity and utility of the TEQ. We believe such studies will advance our understanding of medical student empathy and lead to a more comprehensive understanding and means of enhancing empathy.

## Data Availability

The dataset for the current study is available from the corresponding author on reasonable request.

## Abbreviations

TEQ: Toronto Empathy Questionnaire
JSE: Jefferson Scale of Physician Empathy
IRI: Interpersonal Reactivity Index
CMIN/DF: Minimum Discrepancy per Degree of Freedom
NFI: Normed Fit Index
TLI: Turker Lewis Index
CFI: Comparative Fit Index
RMSEA: Root Mean Square Error of Approximation
